# Detection of maternal carriers of common α-thalassemia deletions from cell-free DNA

**DOI:** 10.1038/s41598-022-17718-7

**Published:** 2022-08-09

**Authors:** Phuoc-Loc Doan, Duy-Anh Nguyen, Quang Thanh Le, Diem-Tuyet Thi Hoang, Huu Du Nguyen, Canh Chuong Nguyen, Kim Phuong Thi Doan, Nhat Thang Tran, Thi Minh Thi Ha, Thu Huong Nhat Trinh, Van Thong Nguyen, Chi Thuong Bui, Ngoc-Diep Thi Lai, Thanh Hien Duong, Hai-Ly Mai, Pham-Uyen Vinh Huynh, Thu Thanh Thi Huynh, Quang Vinh Le, Thanh Binh Vo, Thi Hong-Thuy Dao, Phuong Anh Vo, Duy-Khang Nguyen Le, Ngoc Nhu Thi Tran, Quynh Nhu Thi Tran, Yen-Linh Thi Van, Huyen-Trang Thi Tran, Hoai Thi Nguyen, Phuong-Uyen Nguyen, Thanh-Thuy Thi Do, Dinh-Kiet Truong, Hung Sang Tang, Ngoc-Phuong Thi Cao, Tuan-Thanh Lam, Le Son Tran, Hoai-Nghia Nguyen, Hoa Giang, Minh-Duy Phan

**Affiliations:** 1 Gene Solutions, Ho Chi Minh City, Vietnam; 2 Medical Genetics Institute, Ho Chi Minh City, Vietnam; 3 Hanoi Obstetrics and Gynaecology Hospital, Ha Noi, Vietnam; 4 Tu Du Hospital, Ho Chi Minh City, Vietnam; 5 Hung Vuong Hospital, Ho Chi Minh City, Vietnam; 6 Can Tho Gynaecology and Obstetrics Hospital, Can Tho, Vietnam; 7 Ha Noi Medical University, Ha Noi, Vietnam; 8 University Medical Centre HCM, Ho Chi Minh City, Vietnam; 9 University of Medicine and Pharmacy, Hue University, Hue, Vietnam; 10 Center for Molecular Medicine, University of Medicine and Pharmacy at Ho Chi Minh City, Ho Chi Minh City, Vietnam; 11 Women and Children Hospital of Kien Giang, Kien Giang, Vietnam; 12 Reproductive Health Care Centre of Binh Duong, Binh Duong, Vietnam; 13 Gia Dinh People Hospital, Ho Chi Minh City, Vietnam; 14 Khanh Hoa General Hospital, Nha Trang, Vietnam; 15 Cam Ranh General Hospital, Cam Ranh, Vietnam

**Keywords:** Biotechnology, Computational biology and bioinformatics, Genetics, Diseases

## Abstract

α-Thalassemia is a common inherited blood disorder manifested mainly by the deletions of α-globin genes. In geographical areas with high carrier frequencies, screening of α-thalassemia carrier state is therefore of vital importance. This study presents a novel method for identifying female carriers of common α-thalassemia deletions using samples routinely taken for non-invasive prenatal tests for screening of fetal chromosomal aneuploidies. A total of 68,885 Vietnamese pregnant women were recruited and α-thalassemia statuses were determined by gap-PCR, revealing 5344 women (7.76%) carried deletions including αα/−−^SEA^ (4.066%), αα/−α^3.7^ (2.934%), αα/−α^4.2^ (0.656%), and rare genotypes (0.102%). A two-stage model was built to predict these α-thalassemia deletions from targeted sequencing of the HBA gene cluster on maternal cfDNA. Our method achieved F1-scores of 97.14–99.55% for detecting the three common genotypes and 94.74% for detecting rare genotypes (−α^3.7^/−α^4.2^, αα/−−^THAI^, −α^3.7^/−−^SEA^, −α^4.2^/−−^SEA^). Additionally, the positive predictive values were 100.00% for αα/αα, 99.29% for αα/−−^SEA^, 94.87% for αα/−α^3.7^, and 96.51% for αα/−α^4.2^; and the negative predictive values were 97.63%, 99.99%, 99.99%, and 100.00%, respectively. As NIPT is increasingly adopted for pregnant women, utilizing cfDNA from NIPT to detect maternal carriers of common α-thalassemia deletions will be cost-effective and expand the benefits of NIPT.

## Introduction

Thalassemias occur when the production of hemoglobin is inadequate. As alpha protein is a subunit of hemoglobin, when the production of alpha protein is affected, it results in α-thalassemia. It is reported to be the most common disorder of hemoglobin, impacting 5% of the world’s population, but in Southeast Asia the carrier frequency is reportedly up to 14%^1^. The gene cluster responsible for this disease is located near the tip of chromosome 16 (16p13.3); it is made up from three functional genes in a conserved order 5′–ζ–α_2_–α_1_–3′ (zeta, alpha2, and alpha1). The α_1_ (*HBA1*) and α_2_ (*HBA2*) are nearly identical genes^1^. Most people would effectively have four copies of α genes that produce α-globin polypeptide chains. The predominant α-thalassemia mutations are resulted from deletion of one or more of the four α-globin genes. The two most common α-globin mutations are named rightward and leftward deletions based on the relative positions of misalignment crossovers during meiosis. They are denoted by − α^3.7^ and − α^4.2^ because they result in the deletion of 3.7 and 4.2-kilobase fragments of DNA, respectively^2^. In Southeast Asia, there are other three known, large deletions that are named by geographic regions where they are commonly found. These are −−^SEA^ (Southeast Asia), −−^FIL^ (Philippines) and −−^THAI^ (Thailand), each removes two α-globin genes on the same chromosome (*cis* deletion). Combination of the −−^SEA^, −−^FIL^, or −−^THAI^ deletions with either the −α^3.7^, −α^4.2^ deletions or with a point mutation, are known as “deletional Hb H disease” (missing 3 copies). Consequently, different strategies are built and implemented to detect deletional and non-deletional forms of α-thalassemia. The α^0^-thalassemia (−−^SEA^), and α^+^-thalassemia (−α^3.7^ and −α^4.2^) deletions are of particular importance because they were commonly found in Vietnam^3,4^. Fetuses harboring the homozygous state of α^0^-thalassemia would suffer from a fatal condition known as the hemoglobin (Hb) Bart’s hydrops fetalis syndrome, characterized by severe anemia, hepatosplenomegaly, hypoxia, heart defects, etc. Affected fetuses almost always succumb in utero or die soon after birth^5,6^. In addition, the health of the mother may also be adversely affected. It was estimated that 50% of mothers carrying an affected fetus might suffer lethal complications if they do not receive proper care^2^. When both parents are α^0^-thalassemia carriers, each pregnancy has a 25% risk for Hb Bart’s hydrops.

Generally, an affected fetus can be detected by prenatal diagnosis for at-risk couples by fetal sampling via amniocentesis or chorionic villus sampling (CVS). However, many women are not comfortable with such invasive techniques due to the physical discomfort and the 1% to 2% risk of miscarriage^7^. To eliminate this potential risk, a primary aim in prenatal diagnosis has been to develop non-invasive methods using cell-free DNA (cfDNA) from a maternal blood sample. In recent years, non-invasive prenatal testing (NIPT) using maternal plasma cfDNA has reshaped the existing prenatal care system for pregnancies in terms of screening for common chromosomal aneuploidies^8^. Progress has been made in developing NIPT for monogenic diseases^9^. However, non-invasively detecting deletions such as α-thalassemia deletions for the fetus remains a challenge because fetal DNA represents only a minor fraction of total DNA in maternal plasma^10,11^. An alternative and indirect approach was to screen for carrier status of the pregnant woman to determine the risk of the fetus being born with the disorder. As NIPT is becoming more common for pregnant women, utilizing the maternal cfDNA source from NIPT to detect maternal carriers of common α-thalassemia deletions will be cost-effective and expand the benefits of NIPT.

Multiplex gap polymerase chain reaction (gap-PCR) is a common method to detect common α-thalassemia deletions^12^. However, it is labor intensive, does not work on the same cfDNA sample used for NIPT (ie. requires genomic DNA) and does not scale well to large numbers of samples. Next-generation sequencing (NGS) has been widely used for carrier screening tests for inherited monogenic disorders. However, it remains a challenge to effectively distinguish *HBA1* and *HBA2* genes due to the high homology between them^13,14^. In addition, current NGS based tools are not suitable for detecting copy number variations in the HBA gene cluster due to its low mappability and thus often be excluded from analysis. Therefore, a novel method with the scalability of NGS and is able to utilize cfDNA from NIPT would be needed to provide screening for common α-thalassemia deletions at the same scale as NIPT.

In the present study, we aimed to develop a machine learning model using targeted sequencing by NGS to detect female carriers of common α-thalassemia deletions using samples routinely taken for NIPT purposes. This approach presents two main benefits, eliminating the need for a separate genomic DNA sample and allowing for multiplexing large numbers of samples on one sequencing run. To achieve this, we used a large collection of 68,885 cfDNA samples with known α-thalassemia carrier status from gap-PCR performed on matched genomic DNA samples. With this data set, we have successfully trained an ensemble model consisting of two Random Forest classifiers and achieved highly accurate classification of common α-thalassemia deletions.

## Materials and methods

### Ethical statement and participants

This study was approved by institutional ethics committees of University of Medicine and Pharmacy at HCM city (Approval ID: 164/HDDD). All participants provided signed informed consent. All experimental methods were performed in accordance with relevant guidelines and regulations. All pregnant women who registered for routine NIPT for screening of fetal chromosomal aneuploidies, ages over 18, and agreed to participate in this study were considered eligible. A total of 68,885 Vietnamese pregnant women were recruited between January 2020 and June 2021 from multiple clinics in Vietnam. All samples were processed at the Medical Genetics Institute in Vietnam. The study used the same blood sample drawn for NIPT; no extra sampling was required.

### Sample collection and DNA extraction

Maternal genomic DNA (gDNA) and total cell-free DNA were extracted from whole blood collected in STRECK Blood Collection Tube (STRECK, USA) by venipuncture and fractionated according to manufacturer’s instructions. gDNA and cfDNA were extracted from the buffy coat layer using the MagMAX™ DNA Multi-Sample Ultra 2.0 Kit (ThermoFisher, USA) and the plasma fraction using the MagMAX™ Cell-Free DNA Isolation Kit (ThermoFisher, USA). All extractions were performed on the KingFisher Flex System (ThermoFisher, USA).

### Gap-PCR

Multiplex gap-PCR assays were used to screen for common α-globin deletions (SEA, 3.7, 4.2, THAI, FIL) as previously reported with modifications for touchdown PCR to increase specificity^12^. Each reaction contained 1 mol/L Betaine (Sigma, USA), 0.2 µL of each primer (Supplementary Table S1), 100 ng of genomic DNA and Platinum™ Green Hot Start PCR Master Mix 2X (ThermoFisher, USA) to a 25 µl final volume. The gene LIS1 was co-amplified in each reaction and served as internal control for PCR. Reactions were carried out on a Mastercycler Nexus X2 (Eppendorf, Germany). Touchdown PCR program is presented in Supplementary Table S2. Following amplification, 5 µL of the product was visualized on 1% agarose gel pre-stained with SybrSafe (Invitrogen, USA). Primers and amplicon sizes were summarized in Supplementary Table S1.

### Library preparation for cfDNA and sequencing

NGS library was prepared from cfDNA using NEBNext^®^ Ultra™ II FS DNA Library Prep Kit for Illumina (New England BioLabs, Ipswich, MA, USA) according to the manufacturer’s instructions. DNA library concentrations were quantified with a QuantiFluor^®^dsDNA system (Promega, USA). Equal amounts of libraries (150 ng per sample) were pooled and hybridized with xGen Lockdown probes targeting coding exons of *HBA1* and *HBA2* (IDT DNA, USA). Sequencing was performed using NextSeq 500/550 High output kits v2 (2 × 75 cycles) on the NextSeq 550 system (Illumina, USA) with minimum target coverage of 12X. The mean coverage in the target regions for all samples was approximately 1852X.

### Data preprocessing

The sequencing data from 68,885 cfDNA samples were mapped to the human reference genome (GRCh38) using bwa-mem version 0.7.17^15^ and the number of mapped fragments was counted for 66 bins within 21 kb of the *HBA* gene clusters (see Fig. 2 and Supplementary Table S3) using featureCounts function from Rsubread package version 2.6.0^16^. The raw counts in each bin were then normalized using the Transcripts per million (TPM) method with the following formula TPM = A * (1/(A)) * 10^6, where A = total fragments map to feature * 10^3/feature length in bp^17^.

### Prediction model and statistical analysis

A prediction model was built to classify patients into five groups including αα/αα (healthy), αα/−−^SEA^, αα/−α^3.7^, αα/−α^4.2^ and Others (for rare cases such as −α^3.7^/−α^4.2^, αα/−−^THAI^, −α^3.7^/−−^SEA^, −α^4.2^/−−^SEA^, etc.) The model comprised of two stages, a Random Forest model to perform binary classification (normal/abnormal) followed by another Random Forest model to classify abnormal class into αα/−−^SEA^, αα/−α^3.7^, αα/−α^4.2^ and Others. The model training, validating and testing were performed using a framework provided by the tidymodels packages (ver 0.1.3). Training, validating and testing sets were split 60:20:20 from the full dataset of 68,885 samples. Training data were re-sampled to achieve a balanced data set (3208 normal samples: 3209 abnormal samples containing αα/−α^3.7^ (n = 1052), αα/−α^4.2^ (n = 233), αα/−−^SEA^ (n = 1505) and Rare cases (n = 44)) but validating and testing sets were used at the ratios reflecting the true prevalence of each class in the population. The metrics used to evaluate model performance include Accuracy, F1-Score, Precision, Recall, Confusion matrix, Positive predictive values (PPV) and Negative predictive values (NPV).

**Figure 1:**
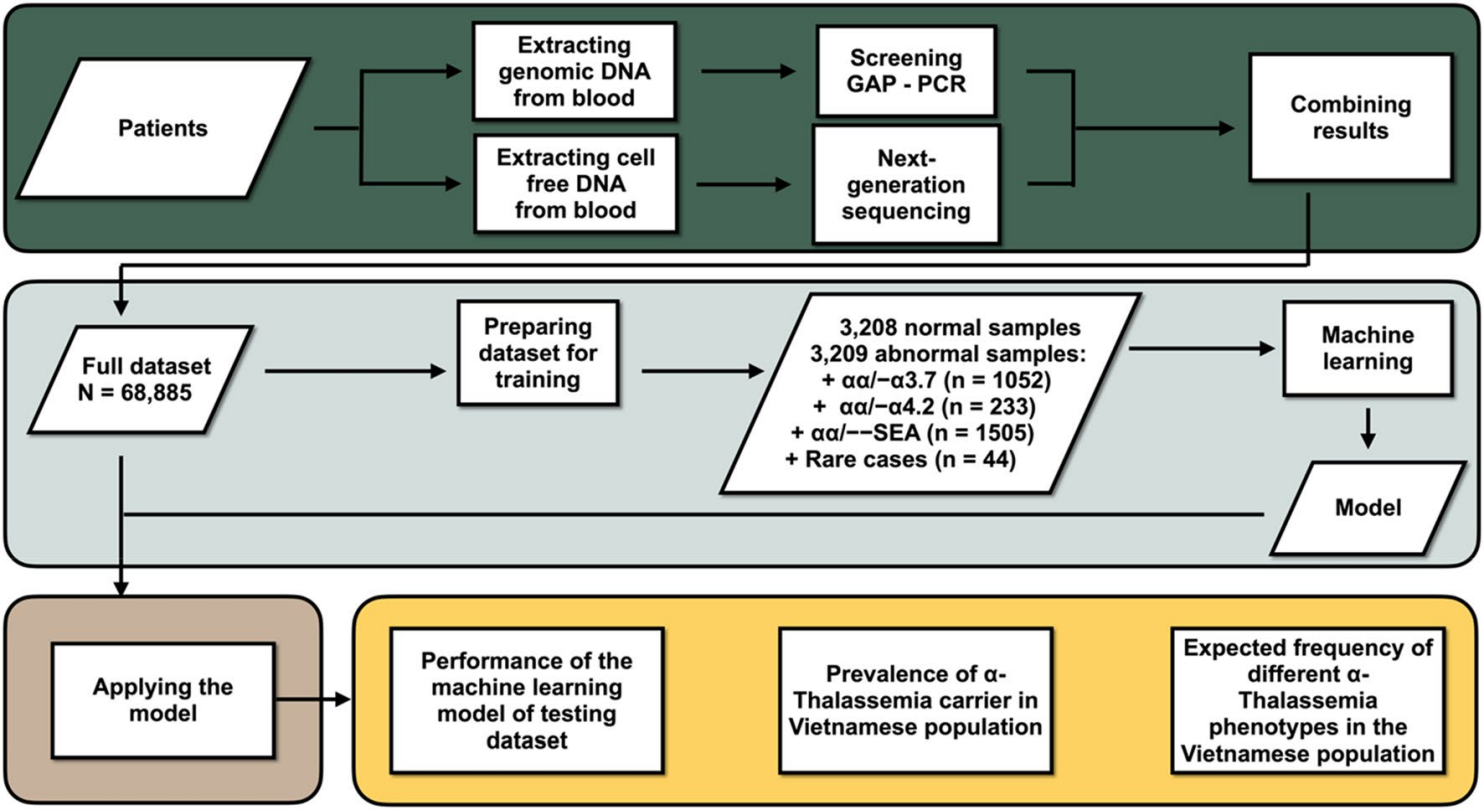
Flow chart of the framework and outcomes.

All data analyses were performed in R version 4.0.1 using packages from CRAN, Tidyverse version 1.3.1, Tidymodels version 0.1.3 for building models, Gviz version 1.32.0 and ggplot2 version 3.3.3 for plotting, caret version 6.0–88 for attaining performance metrics^18–22^. A flow chart providing an overview of workflow is presented in Fig. 1.

Data analysis was performed using descriptive statistics (i.e. frequencies or medians). Punnett Square was used to estimate population genotype and phenotype frequency^23^.

## Results

### α-Globin genotype distribution in our study cohort

**Figure 2:**
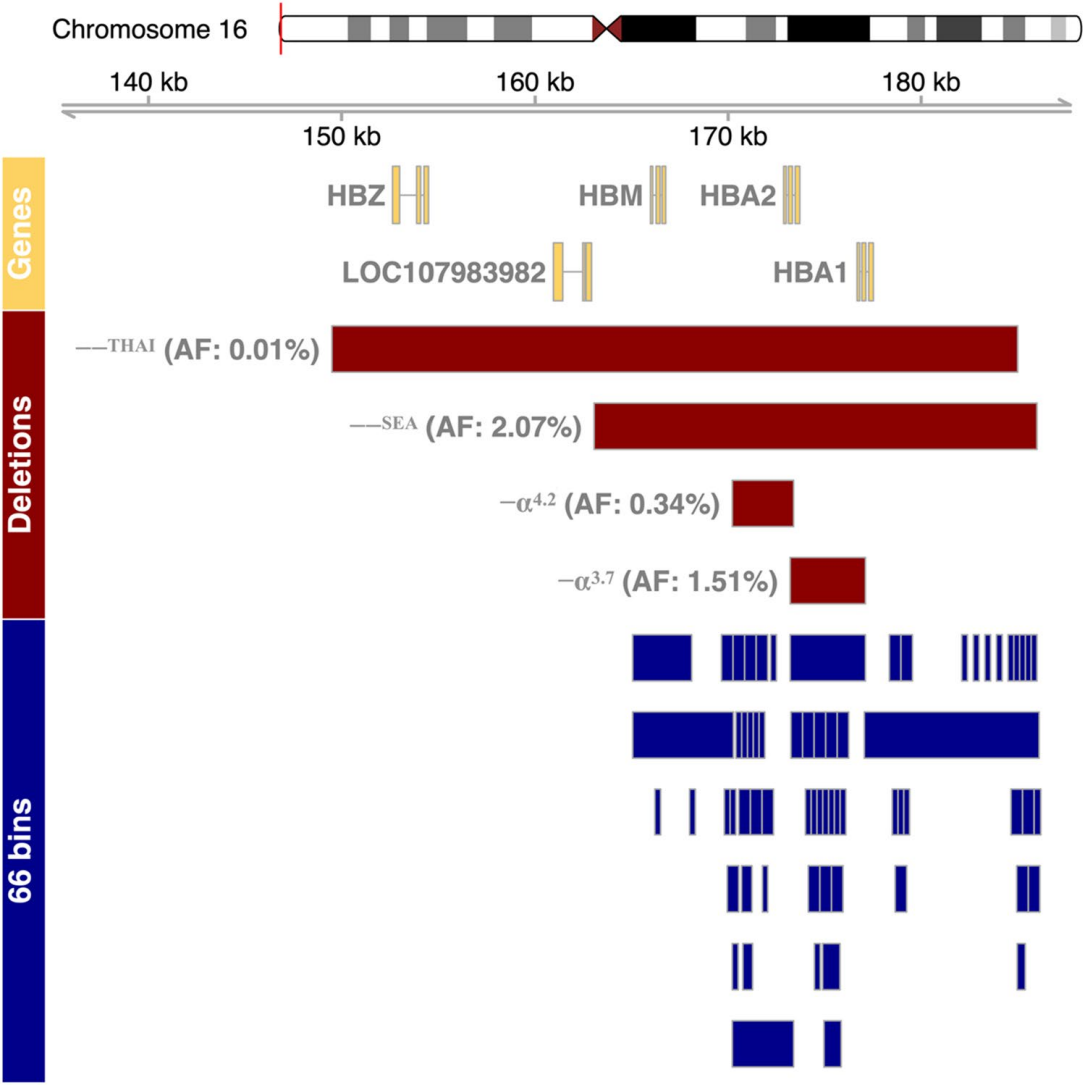
The human α-globin gene cluster and the mutations detected. The Genes track panel illustrates the α-globin locus on the tip of chromosome 16. The approximate ranges of the four deletions (red bar) detected in this study population (−−^SEA^, −α^3.7^,  α^4.2^ and −− ^THAI^) are shown in the “Deletions” track. The ranges of 66 bins (blue bars) within the α-globin gene cluster are shown in the “66 bins” track. The counts of fragments mapped to each bin were used as the raw data for our prediction model. −−^SEA^, Southeast Asian deletion; −−^THAI^, Thailand deletion; AF, Allele frequency. The figure was drawn using the Gviz package.

The α-globin genotypes of all samples as determined by gap-PCR are presented in Table 1. Deletion mutations of the α-globin gene cluster were detected in 5344 of 68,885 individuals (7.76%), of which the Southeast Asian type αα/−−^SEA^ was the most common deletional genotype accounting for 4.066% (n = 2801) of all cases. The −−^SEA^ deletion was also found in forty-nine individuals who had compound heterozygous genotypes of −α^3.7^/−−^SEA^ and seven of −α^4.2^/−−^SEA^. The second most common genotype was the −α^3.7^/αα, with a prevalence of 2.934% (n = 2021), followed by −α^4.2^/αα at 0.656% (n = 452). The genotype of the *trans* form of α-thalassemia trait (−α^3.7^/−α^4.2^) was also found in the study population in six cases. Eight individuals were found to be of genotype αα/−−^THAI^. A diagram illustrating the four deletion mutations (−^SEA^, −α^3.7^, −α^4.2^ and −−^THAI^) found in our cohort and their allele frequencies was summarised in Fig. 2.

**Table 1:** α-Globin genotypes in the study population.

α-Globin genotypes	Phenotypes	No. of cases	Frequency (%)
**Common Genotypes**		**68,815**	**99.898**
αα/αα	Normal	63,541	92.242
(αα/−−^SEA^)	α-Thalassemia trait	2801	4.066
(−α^3.7^/αα)	α-Thalassemia silent carrier	2021	2.934
(−α^4.2^/αα)	α-Thalassemia silent carrier	452	0.656
**Rare Genotypes**		**70**	**0.102**
(−α^3.7^/−−^SEA^)	Hb H disease	49	0.071
(αα/−−^THAI^)	α-Thalassemia trait	8	0.012
(−α^4.2^/−−^SEA^)	Hb H disease	7	0.010
(−α^3.7^/−α^4.2^)	α-Thalassemia trait	6	0.009

Total values for Common Genotypes and Rare Genotypes are in [bold].

### An ensemble model to classify common α-globin genotypes from cfDNA data

**Figure 3:**
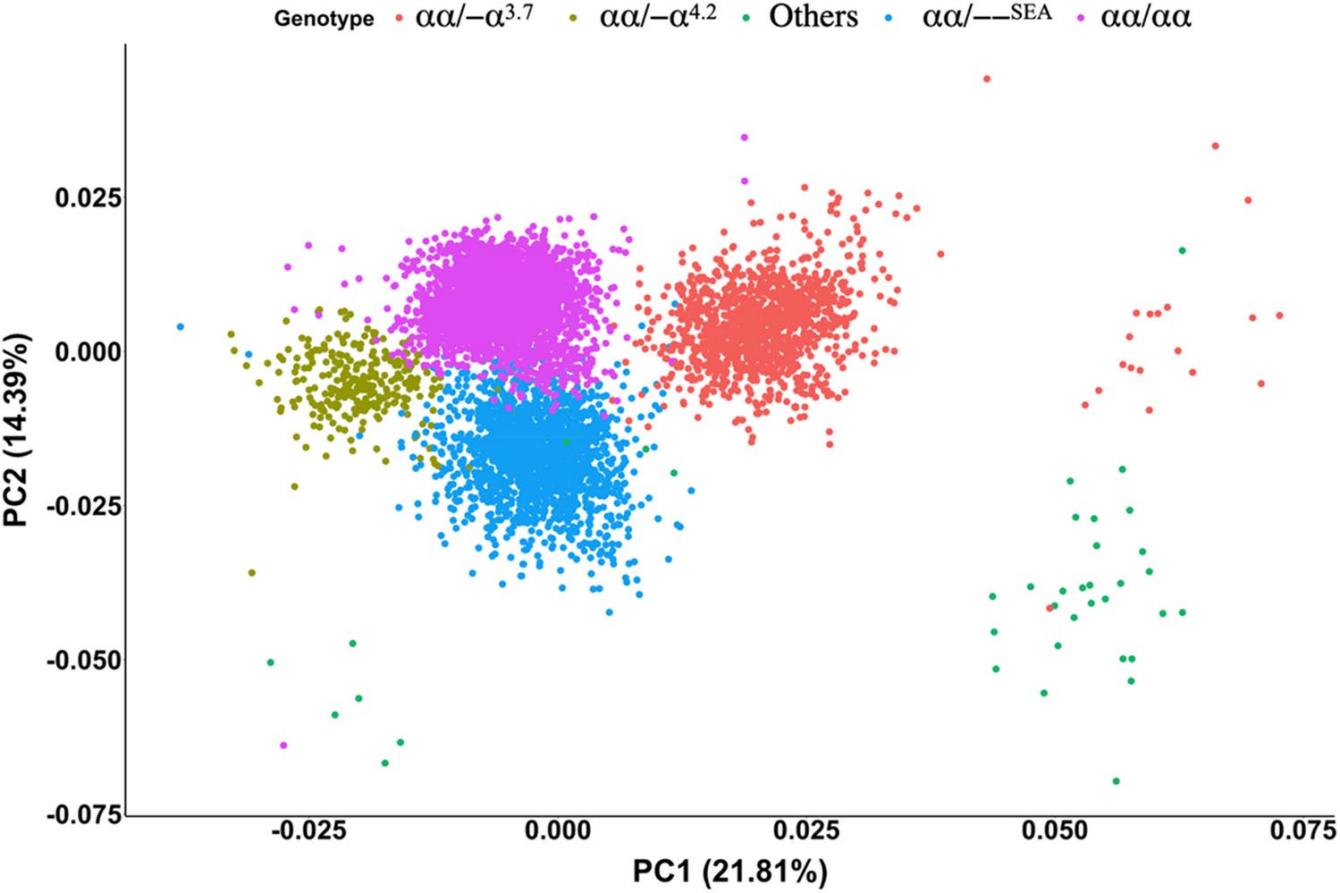
Principal component analysis of 6417 samples and their genotypes. Normalized count data of 66 features (bins) from 6417 samples within the training dataset were used for principal component analysis. Only PC1 and PC2 are shown along with the percent of variation explained by each component in brackets. Each dot represents one sample.

Paired-end sequencing was performed on cfDNA obtained from 68,885 samples (Table 1). The sequencing data were mapped, sorted and converted into input features by counting the number of fragments overlapping each of the 66 bins within the ~ 21 kb of the *HBA* gene cluster (Fig. 2) and then normalized to account for feature length and sequencing depth. The data were then split into training, validating and testing sets with 60:20:20 ratio. A summary of the training dataset using principal component analysis was presented in Fig. 3, demonstrating that the clustering of samples fit nicely with their genotypes.

Although our main focus was the classification of 4 common genotypes αα/αα, αα/−−^SEA^, αα/−α^3.7^ and αα/−α^4.2^, we noticed that the rare genotypes accounted for 0.102% of our samples (Table 1). We believed this was a significant number that should not be ignored. This is because affected people are at increased risk for having children with α-thalassemia major. Therefore, we decided to build an ensemble model which classifies the data over two stages, with a Random Forest classifier used in each stage; the first stage aimed to separate normal (αα/αα) from abnormal cases, while the second stage was built to classify the abnormal cases into αα/−−^SEA^, αα/−α^3.7^, αα/−α^4.2^ or Others (including all rare genotypes).

### Model performance

The ensemble model achieved excellent performance on the testing set of 13,777 samples, with the overall accuracy of 99.78% (Table 2). The binary classification stage showed 100% sensitivity and 100% NPV for the abnormal class, demonstrating a performance with minimal false negatives for all abnormal samples including those with rare genotypes. This stage therefore had fulfilled its designed intention of detecting common as well as rare α-thalassemia deletional genotypes. In the ensemble model, the αα/αα class, which made up 92.23% of the testing set, had an F1-score of 99.90%, PPV of 100% and NPV of 97.63%. The common genotypes including the αα/−−^SEA^, αα/−α^3.7^ and αα/−α^4.2^ classes, which together account for 7.63% of the testing set, showed the F1-scores in the range of 97.14–99.55%. The “Others” (rare genotypes) class had an F1-score of 94.74%, which was the lowest among the classes. However, this low F1-score was the result of misclassification of these rare genotypes into one of the common genotypes but not the normal genotypes. Although these were misclassifications, the consequences were less severe than a misclassification into the αα/αα class. In summary, the ensemble model successfully classified α-thalassemia carriers using data from cfDNA samples with high performance.

**Table 2:** Performance metrics of the model on the testing dataset (total case number = 13,777).

Genotypes	No. of cases	Accuracy	F1 (%)	Sensitivity (%)	Specificity (%)	PPV (%)	NPV (%)
**Stage 1**							
Abnormal	1070	99.81%	98.80	100.00	99.80	97.63	100.00
**Ensemble**							
αα/αα	12,707	99.78%	99.90	99.80	100.00	100.00	97.63
αα/−−^SEA^	559		99.55	99.82	99.97	99.29	99.99
αα/−α^4.2^	83		98.22	100.00	99.98	96.51	100.00
αα/−α^3.7^	409		97.14	99.51	99.84	94.87	99.99
Others	19		94.74	94.74	99.99	94.74	99.99

### Estimated frequency of affected individuals

**Figure 4:**
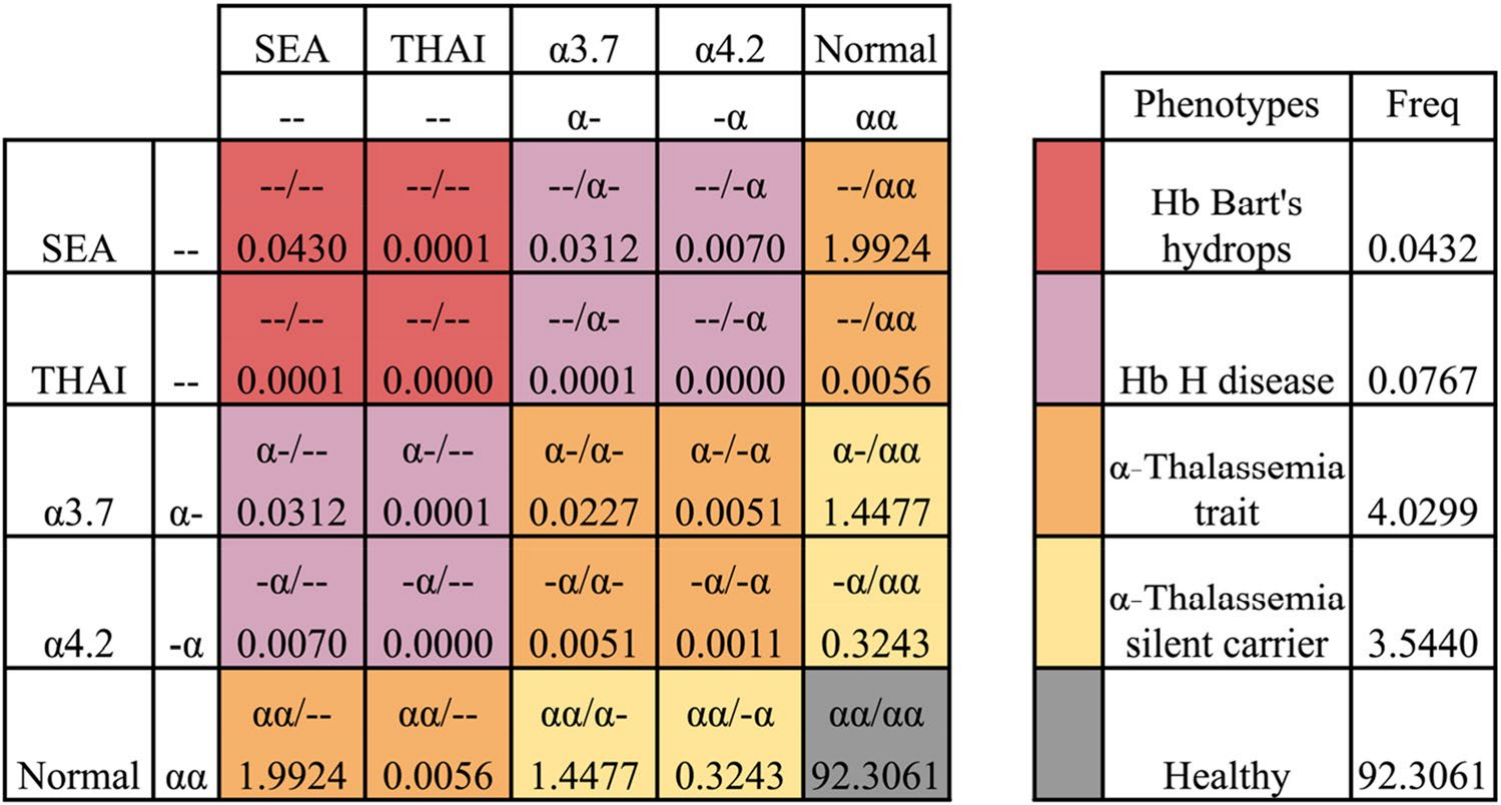
Estimated incidence (%) of α-thalassemia phenotypes in the Vietnamese population.

There exists an increasing need for a greater understanding of the epidemiology of α- thalassemia in order that burden can be determined to guide public health decisions and assess the need for genetic counselling and treatments. However, it remains challenging due to the lack of information on the prevalence, biodiversity and health burden of α-thalassemia in Vietnam. Using the observed genotype data and assuming Hardy–Weinberg equilibrium, Punnett square calculation was employed to determine expected frequency of different α-thalassemia phenotypes in the Vietnamese population. These calculations revealed that the expected frequencies of Hb Bart's hydrops, Hb H disease, α-thalassemia trait and α-thalassemia silent carrier were 0.0432%, 0.0767%, 4.0299% and 3.5440%, respectively (see Fig. 4). Given the number of children born in 12 months prior to 01/4/2019 in Vietnam, which was 1,394,401 births according to the Completed results of the 2019 Vietnam population and housing census, these frequencies would translate into 603, 1070, 56,193 and 49,418 babies born with Hb Bart's hydrops, Hb H disease, α-thalassemia trait and α-thalassemia silent, respectively, if no action is taken^24^. Therefore, a prenatal screening programme using our ensemble model would potentially identify and provide proper care for 106,686 pregnant women per year with regards to α-thalassemia in Vietnam.

## Discussion

α-Thalassemia is a recessive hereditary disease that can manifest into moderate-to-severe forms in individuals with combined or homozygous mutations, resulting in health problems such as anemia, growth delays, and hemolysis, and some people with α-thalassemia may require chronic blood transfusions. The costs of medical treatment for α-thalassemia and other hemoglobin-related diseases can be a huge burden for the healthcare system in countries with high prevalence, as much as $220 million per year in Thailand or $200 million per year in Iran^25,26^. Therefore, screening programs are needed to properly manage severe cases of α-thalassemia, especially in countries with high prevalence of carriers such as Vietnam. The advent of NIPT opened up a new era of using non-invasive approaches to screen for genetic anomalies in the fetus. However, detecting α-thalassemia deletion in the fetus using cell-free DNA remains a challenge. It is therefore necessary to determine the carrier status of the mother (and subsequently the father if the mother is indeed a carrier) in order to calculate the risk of a fetus developing α-thalassemia. Here we present a protocol using cell-free DNA from maternal blood to screen for α-thalassemia carrier status in the mother. This protocol was designed to complement NIPT and did not require a separate blood sample. The same vial of cell-free DNA was prepared into a NGS library for NIPT, then an aliquot of this library was used for target capture and sequencing of the HBA gene cluster. A machine learning model was then used to predict the carrier status. We believe this is an accurate and cost-effective approach that can be used for all pregnant women undergoing NIPT.

In this work, we presented an approach using fragment count profiles across the HBA gene cluster (~ 21 Kb) to detect α-thalassemia deletions by utilizing cfDNA from maternal blood samples. We demonstrated that it was possible to take a machine learning-based approach to classify a cfDNA profile into α-thalassemia carrier status. Our model achieved the sensitivity and specificity of more than 99% for all common genotype classes (i.e., αα/−−^SEA^, αα/−^α3.7^ and αα/−α^4.2^). The Southeast Asian deletion (−^SEA^) is the most common and severe form of α-thalassemia that was not only found in Southeast Asia and South China^25,27^ but also in our data at 4.07%. We determined the PPV and NPV of our model for this genotype (αα/−−^SEA^) as 99.29% and 99.99%, respectively. The PPV of our model was superior to that of the IC strip assay with a reported PPV of 51.2% and a comparable NPV of 100% ^28^. Additionally, the model could also detect all the rare genotypes that were found in our data with reasonable sensitivity of 94.74% and a nearly perfect specificity of 99.99%. This included the detection of αα/−−^THAI^ which is thought to be very rare in the Vietnamese population. Attempts to detect Hb Bart’s of the fetus directly from maternal cfDNA were made using cycle threshold cut-off from real time quantitative PCR. Despite the promising results, this method was limited to only Hb Bart’s in the fetus with 98.4% sensitivity and 20.8% false-positive rate^11^. Even though our model could not directly detect the HBA genotype of the fetus, these results suggested our machine learning-based test could be used in α-thalassemia carrier screening programs for pregnant women in Vietnam.

To apply our test in clinical practice, we propose a routine procedure where the fetal chromosomal aneuploidies and maternal HBA genotype are simultaneously examined using a single blood draw. If the mother is a carrier, HBA genotyping of the father is offered. When both the mother and the father are found to be carriers, genetic counselling is offered to discuss further assessment, which might include invasive test to directly confirm the HBA genotype of the fetus. If used routinely, this procedure will provide assurance for 92% of pregnant women that their child has no risk of α-thalassemia while identifying the remaining female carriers that would be benefited from extra care and counselling. While accessing the economic benefit of such procedure is beyond the scope of this study, we believe that it is easier to scale up than standard hemoglobin electrophoresis or gap-PCR method, and thus can meet the demand of pregnant women for better assessment of the α-thalassemia risk in their developing child.

To the best of our knowledge, this work represents the largest study to report the prevalence of α-thalassemia in Vietnam. We used the data to estimate the burden of severe forms of α-thalassemia caused by deletion mutations. Approximately 106,681 fetuses were estimated to be affected by Hb H disease, α-thalassemia trait or α-thalassemia silent. An example of a successfully implemented carrier screening program was in Sardinia, Italy. The voluntary carrier screening programme was effective, as indicated by the decreasing of the birth rate of thalassemia major from 1:250 to 1:4000 in the 20 years of the program^29^. We hope that by adopting this machine learning-based test, we can significantly reduce the incident rate of severe α-thalassemia in Vietnam in the future.

This study has several limitations. First, although Vietnam has 54 ethnic groups (excluding foreign immigrants), our study did not collect ethnicity information of the participants. Therefore, the prevalence reported here did not reflect the prevalence of any particular ethnic group in Vietnam. Second, the data used to train our model were based on gap-PCR, therefore the performance of our model might be constrained by the inherence accuracy of gap-PCR. Further study to investigate cases with discordance results between our model and gap-PCR using a third method might be needed to better calculate its performance metrics.

In summary, this study presents a novel screening test using maternal cfDNA and a machine learning model to detect maternal carriers of common α-thalassemia deletions. The use of maternal cfDNA allows this test to be included as an extension of routine non-invasive prenatal testing for chromosomal anomalies without a separate blood sample. For a population with high prevalence of α-thalassemia such as Vietnam this test would benefit hundreds of thousands of women and their children per year.

## Supplementary Information

Supplementary Information.

## Data Availability

The data that support the findings of this study are available from the corresponding author upon reasonable request. The data are not publicly available due to privacy or ethical restrictions.
