# Supplementary material for: Detection of maternal carriers of common α-thalassemia deletions from cell-free DNA
Source: Sci Rep. 2022 Aug 9;12:13581. doi: 10.1038/s41598-022-17718-7 (PMC9363435; doi:10.1038/s41598-022-17718-7)
Supplement: Supplementary file 1 — Supplementary Information. [file 41598_2022_17718_MOESM1_ESM.pdf]

Detection of maternal carriers of common α-thalassemia deletions from cell-free DNA

Phuoc-Loc Doan, Duy-Anh Nguyen, Quang Thanh Le, Diem-Tuyet Thi Hoang, Huu Du Nguyen, Canh Chuong Nguyen, Kim Phuong Thi Doan, Nhat Thang Tran, Thi Minh Thi Ha, Thu Huong Nhat Trinh, Van Thong Nguyen, Chi Thuong Bui, Ngoc-Diep Thi Lai, Thanh Hien Duong, Hai-Ly Mai, Pham-Uyen Vinh Huynh, Thu Thanh Thi Huynh, Quang Vinh Le, Thanh Binh Vo, Thi Hong-Thuy Dao, Phuong Anh Vo, Duy-Khang Nguyen Le, Ngoc Nhu Thi Tran, Quynh Nhu Thi Tran, Yen-Linh Thi Van, Huyen-Trang Thi Tran, Hoai Thi Nguyen, Phuong-Uyen Nguyen, Thanh-Thuy Thi Do, Dinh-Kiet Truong, Hung Sang Tang, Ngoc-Phuong Thi Cao, Tuan-Thanh Lam, Le Son Tran, Hoai-Nghia Nguyen, Hoa Giang, Minh-Duy Phan

Table S1. Primers for multiplex PCR screening for common α-globin deletions

Primer ID	Sequence (5'-3')	Amplicon size (bp)
**LIS-F**	GTCGTCACTGGCAGCGTAGATC	2503
**LIS-R**	GATTCCAGGTTGTAGACGGACTG	
**3.7-F**	CCCCTCGCCAAGTCCACCC	2022
**3.7-R**	AAAGCACTCTAGGGTCCAGCG	
**4.2-F**	GGTTTACCCATGTGGTGCCTC	1628
**4.2-R**	CCCGTTGGATCTTCTCATTTCCC	
**SEA-F**	CGATCTGGGCTCTGTGTTCTC	1349
**SEA-R**	AGCCCACGTTGTGTTCATGGC	
**THAI-F**	TGACTGCATCATAATTCCAGCAG	480
**THAI-R**	TGAGGCAGGAGATTCGCTTGA	
**FIL-F**	CTGCCCTTCACACCTCAGACA	597
**FIL-R**	GCAATCTTGGCTCACTGCAGG	

Table S2. Touchdown PCR program

Temperarute	Time	Step (cycle)
95˚C	5m	1
95˚C	45s	15<br>Touchdown PCR<br>(*): -0.5 ˚C each cycle
68˚C -59˚C (*)	1m15s	
72˚C	2m	
95˚C	45s	15
59˚C	1m15s	
72˚C	2m	
72˚C	10m	1
4˚C	hold	1

Table S3. The ranges of 66 bins within the α-globin gene cluster

GeneID	Chr	Start	End
B16	chr16	169801	170100
B17	chr16	170101	170400
B20	chr16	171001	171300
B30	chr16	174001	174300
B34	chr16	175201	175500
B35	chr16	175501	175800
B36	chr16	175801	176100
C16	chr16	169651	170250
C17	chr16	169951	170550
C20	chr16	170851	171450
C23	chr16	171751	172350
C30	chr16	173851	174450
C31	chr16	174151	174750
C32	chr16	174451	175050
C33	chr16	174751	175350
C34	chr16	175051	175650
C35	chr16	175351	175950
C36	chr16	175651	176250
K7	chr16	170212	170517
K16	chr16	170762	171266
K24	chr16	171776	172053
K34	chr16	174468	174772
K37	chr16	174963	175857

B4	chr16	166201	166500
B10	chr16	168001	168300
B18	chr16	170401	170700
B19	chr16	170701	171000
B21	chr16	171301	171600
B22	chr16	171601	171900
B24	chr16	172201	172500
B31	chr16	174301	174600
B32	chr16	174601	174900
B33	chr16	174901	175200
B45	chr16	178501	178800
B46	chr16	178801	179100
B47	chr16	179101	179400
B57	chr16	182101	182400
B59	chr16	182701	183000
B61	chr16	183301	183600
B63	chr16	183901	184200
B65	chr16	184501	184800
B66	chr16	184801	185100
B67	chr16	185101	185400
B68	chr16	185401	185700
B69	chr16	185701	186000
C18	chr16	170251	170850
C19	chr16	170551	171150
C21	chr16	171151	171750

C22	chr16	171451	172050
C28	chr16	173251	173850
C45	chr16	178351	178950
C46	chr16	178651	179250
C47	chr16	178951	179550
C66	chr16	184651	185250
C67	chr16	184951	185550
C68	chr16	185251	185850
C69	chr16	185551	186150
C70	chr16	185851	186200
SEA_1	chr16	165053	170273
SEA_1_1	chr16	165053	168113
HBA42	chr16	170213	173393
HBA42_1	chr16	170694	171233
HBA37	chr16	173213	177113
HBA37_1	chr16	174894	175793
SEA_2	chr16	177053	186113
SEA2_1	chr16	184974	185393
